# Assessing the effect of a partly unobserved, exogenous, binary time-dependent covariate on survival probabilities using generalised pseudo-values

**DOI:** 10.1186/s12874-017-0430-5

**Published:** 2018-01-19

**Authors:** Ulrike Pötschger, Harald Heinzl, Maria Grazia Valsecchi, Martina Mittlböck

**Affiliations:** 1 Children’s Cancer Research Institute, A-1090 Vienna, Austria; 2 Center for Medical Statistics, Informatics, and Intelligent Systems, Medical University of Vienna, Spitalgasse 23, A-1090 Vienna, Austria; 3 Center of Biostatistics for Clinical Epidemiology, Department of Health Sciences, University of Milan-Bicocca, Piazza dell’Ateneo Nuovo, 1, I-20126 Milan, Italy

**Keywords:** Cox model with a time dependent covariate, Cumulative hazard ratio, Genetic randomization, Non-proportional hazards, Stem cell transplantation, Waiting time bias

## Abstract

**Background:**

Investigating the impact of a time-dependent intervention on the probability of long-term survival is statistically challenging. A typical example is stem-cell transplantation performed after successful donor identification from registered donors. Here, a suggested simple analysis based on the exogenous donor availability status according to registered donors would allow the estimation and comparison of survival probabilities. As donor search is usually ceased after a patient’s event, donor availability status is incompletely observed, so that this simple comparison is not possible and the waiting time to donor identification needs to be addressed in the analysis to avoid bias. It is methodologically unclear, how to directly address cumulative long-term treatment effects without relying on proportional hazards while avoiding waiting time bias.

**Methods:**

The pseudo-value regression technique is able to handle the first two issues; a novel generalisation of this technique also avoids waiting time bias. Inverse-probability-of-censoring weighting is used to account for the partly unobserved exogenous covariate donor availability.

**Results:**

Simulation studies demonstrate unbiasedness and satisfying coverage probabilities of the new method. A real data example demonstrates that study results based on generalised pseudo-values have a clear medical interpretation which supports the clinical decision making process.

**Conclusions:**

The proposed generalisation of the pseudo-value regression technique enables to compare survival probabilities between two independent groups where group membership becomes known over time and remains partly unknown. Hence, cumulative long-term treatment effects are directly addressed without relying on proportional hazards while avoiding waiting time bias.

**Electronic supplementary material:**

The online version of this article (10.1186/s12874-017-0430-5) contains supplementary material, which is available to authorized users.

## Background

Evaluating the effect of a partly unobserved, exogenous, binary time-dependent covariate on long-term survival probabilities is statistically demanding in particular if non-proportional hazards are present. All these challenges are comprised in the motivating example from paediatric oncology.

Although survival after childhood leukaemia has greatly improved over the last decades, there are still subgroups of patients where conventional chemotherapy leads to poor survival outcome. For these high-risk patients, more intense therapies are needed and allogeneic stem cell transplantation (SCT) is often considered a therapeutic option compared to conventional chemotherapy. Due to higher treatment intensity and the graft versus leukaemia effect, SCT may be more efficient in preventing disease recurrences. Contrary, higher rates of early treatment related mortality have to be anticipated with SCT. Hence, it is nearly certain that the hazards are increasing shortly after SCT compared to continuous conventional chemotherapy and decreases over time, expectedly below hazards of continuous conventional chemotherapy. Consequently, non-proportional hazards are frequently observed in studies where SCT is compared with ongoing chemotherapy [1–3]. Patients usually enter a study, when they are considered to be eligible for SCT. Although donor search is initiated immediately thereafter, it can take several months before a donor is found and SCT can be performed. In the meantime, patients are treated with conventional chemotherapy. If no matched donor can be found, conventional chemotherapy remains the only administered treatment. Typically, the effect on the proportion of long-term survivors (cured patients) is most interesting in paediatric oncology studies.

The so-called genetic randomisation approach has been suggested to analyse SCT trials [3–5]. The efficacy of the treatment options “potential SCT after chemotherapy” and “chemotherapy alone” is assessed by simply comparing the long-term survival of those patients who have a donor available and can potentially receive an SCT with those who do not have a suitable donor and for whom SCT is impossible. If actually performed SCT is used as treatment indicator, then waiting time (immortal time) and selection bias will be introduced. Genetic randomisation follows the ideal of the intention-to-treat approach in randomised trials by using the presence or absence of a donor as a surrogate for randomized treatment options. However, its validity requires that both, donor availability status and time until donor identification are independent of patient’s prognosis. These assumptions are usually justified in the context of SCT in childhood leukaemia, where donor availability status only depends on HLA-type which is measured at baseline and is assumed to be independent on the current and future disease state of the patient. Genetic randomisation can straightforwardly be applied if donor availability status is observed for all patients. However, it is common – in particular in studies with matched unrelated donors - that for financial and ethical reasons donor search is ceased after a patient has died. Donor availability status remains unobserved for these patients and genetic randomisation cannot be used anymore.

In this situation, a statistical approach which considers the waiting time to donor availability has to be applied. Currently, Cox regression with a binary time-dependent covariate and landmark analysis are frequently applied. However, with the anticipated non-proportional hazards, the Cox model with a time-dependent covariate is hardly able to provide conclusive information on long-term survival treatment effects. Ignoring time-dependent covariates for the moment, extensions of the Cox-model to deal with non-proportional hazards, i.e. weighted approaches [6–8] or extensions considering time-varying hazard ratios [9–13] do not address long-term survival. These approaches investigate the relative instantaneous risks, i.e. the weighted average of hazard ratios over time or the variation of hazard ratios in time. With non-proportional hazards the interpretation of weighted approaches remains ambiguous; neither does a weighted hazard ratio in favour of an experimental arm automatically imply better long-term survival, nor does a weighted hazard ratio of one necessarily imply that long-term survival probabilities are unaffected by therapy. With time-varying hazard ratios, the hazard ratio at a specific time is conditional on being at risk at this time, which addresses a question that differs from the primary interest in cumulative survival effects [14, 15]. Consequently, extending these approaches to allow for time dependent covariates will not work as well.

The second approach, landmark analysis [16–19] and its extension dynamic prediction by landmarking [20] is mainly used to estimate survival probabilities and to graphically represent survival curves. Here, a later starting point for survival, the landmark time, is arbitrarily chosen and the interpretation of landmark results is naturally hampered by the intrinsic conditioning on being alive at the landmark time and ignoring potential important information before the landmark time point.

In particular with non-proportional hazards, it is crucial to directly address the primary interest on long-term survival [14] either by testing for differences in estimated survival probabilities [15] or by using pseudo-values [14], both for a pre-specified long-term time point. When survival curves reach a plateau, cure rate models [21] are another sensible alternative that allows to compare the probabilities of ‘cured’ long-term survivors without the need to pre-specify a long-term time-point.

Survival probabilities are directly linked to cumulative hazards (minus-log-survival). Consequently, the ratio of the cumulative hazard functions at a pre-specified time point is an alternative natural choice to quantify and compare treatment effects [22–24] with the straightforward interpretation that a cumulative hazard ratio below and above one implies higher and lower long-term survival probabilities, respectively. The concept of cumulative hazard ratios is naturally linked to the recently developed pseudo-value regression technique [25–30] for censored survival data. However, the original pseudo-value regression technique as well as other techniques that directly address long-term survival rates [15, 21] do not allow for time-dependent covariates. Hence, a generalisation to evaluate and compare cumulative treatment effects in the presence of an exogenous binary time-dependent covariate is introduced here. This generalised approach mimics the intention-to-treat analysis of a genetic randomisation in an SCT trial where donor availability is partly unknown. In the following methods sections the new approach is presented, followed by a simulation study that elaborates its properties. Subsequently, the novel approach is applied to real data in paediatric oncology. The discussion section summarises the properties of the new approach and critically outlines its advantages and disadvantages.

## Methods

### The compartment representation for transplant data

By focussing on cumulative treatment effects and prediction of survival probabilities as primary aim, the most interesting outcome of such studies is survival at a pre-specified long-term time point ${t}^{\ast }$, e.g., in order to investigate 5-years survival probabilities, ${t}^{\ast }$ would be set equal to 5 years. In line with the approach of genetic randomisation, two separate populations have to be distinguished conditional on their donor availability status, and survival probabilities in patients with and without a donor available, ${S}_1\left({t}^{\ast}\right)$ and ${S}_0\left({t}^{\ast}\right)$, have to be compared, respectively. Donor availability is defined as the identification of a donor in the time interval from study entry to maximum donor search time ${t}^{search}$, with ${t}^{search}\le {t}^{\ast }$.

### Survival in patients with donor available

At first, survival ${S}_1\left({t}^{\ast}\right)$ in patients with an available donor is investigated. Let $W$ denote a random variable representing the (waiting) time from a given origin (time 0) to the time when a donor is identified, $0\le W\le {t}^{search}$. The distribution of $W$ is characterised by the density ${f}_{01}(t)$ with corresponding hazard function ${\lambda}_{01}(t) dt=P\left(W\in \left[t,t+ dt\right)|W\ge t\right)$. In theory, $W$ can always be observed for this population independently of the survival or censoring status of the patients by prolonging donor search until ${t}^{search}$. In practice, as outlined above, donor availability remains unobserved in patients that die or become censored before donor identification.

**Fig. 1:**
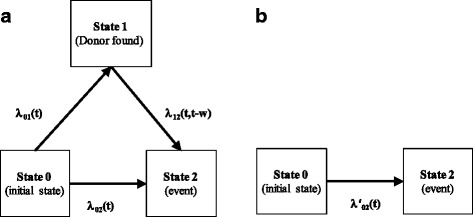
Stochastic process in the two populations with (**a**) and without (**b**) donor available, $w$ is time of transition to state 1. In **a**, all patients have a donor and move from state 0 either to state 1 or state 2 until ${t}^{search}$

Survival in the population with available donor corresponds to a stochastic process with three states (Fig. 1a), where all patients start at the initial state 0.

Let $T$ denote the failure time to reach state 2. The absorbing state 2 can be reached either directly (0→2) if $T<W$ or through the intermediate state 1 (0→1→2) if $T\ge W$. Let ${\lambda}_{02}(t)$ denote the hazard function for a transition at time $t$ from the initial state 0 direct to the absorbing state 2. Accordingly

$$ {\lambda}_{02}(t) dt=P\left(T\in \left[t,t+ dt\right)|T\ge t,W>t\right) $$

Note that in patients with a donor (Fig. 1a), no patients remain in state 0 at ${t}^{search}$ as all patients have either moved to state 1 or to state 2 until ${t}^{search}$.

Let ${\lambda}_{12}\left(t,t-w\right)$ denote the hazard function for a transition from the transient state 1 to the absorbing state 2 which may depend on both, time since start, $t$, and time since transition to state 1, $t-w$.

$$ {\lambda}_{12}\left(t,t-w\right) dt=P\left(T\in \left[t,t+ dt\right)|T\ge t,W=w,t\ge w\right) $$

Note that donor availability can be considered an external (exogenous) stochastic process with

$$ P\left(T\in \left[u,u+ du\right)|X(u),T\ge u\right)=P\left(T\in \left[u,u+ du\right)|X(t),T\ge u\right) $$

for all $u,t$ such that $0<u\le t$ and $X(t)=\left\{x(u);0\le u<t\right\}$ denotes the covariate history of the external time-dependent covariate $x(t)$; for details see p.196 of Kalbfleisch and Prentice [31].

Denote

1 $$ {S}_1\left({t}^{\ast }|w\right)=\exp \left[-\left\{\underset{0}{\overset{w}{\int }}{\lambda}_{02}(v) dv+\underset{w}{\overset{t^{\ast }}{\int }}{\lambda}_{12}\left(v,v-w\right) dv\right\}\right] $$

as survival in a population with a fixed waiting time $W=w$. That is, before transition to state 1 the hazard function ${\lambda}_{02}(t)$ applies and ${\lambda}_{12}\left(t,t-w\right)$ thereafter. Survival in the population with a donor is then

2 $$ {\displaystyle \begin{array}{c}{S}_1\left({t}^{\ast}\right)=\underset{0}{\overset{t^{\ast }}{\int }}{\lambda}_{01}(w)\exp \left[-\underset{0}{\overset{w}{\int }}{\lambda}_{01}(v) dv\right]\left[\exp \left\{-\underset{0}{\overset{w}{\int }}{\lambda}_{02}(v) dv-\underset{w}{\overset{t^{\ast }}{\int }}{\lambda}_{12}\left(w,v\right) dv\right\}\right] dw\\ {}=\underset{0}{\overset{t^{\ast }}{\int }}{f}_{01}(w)\;{S}_1\left({t}^{\ast }|w\right) dw\end{array}} $$

### Survival in patients without donor available

In a genetic randomisation study, patients without a donor available until ${t}^{search}$ form the control group. Let ${T}^{\hbox{'}}$ denote the failure time in a classical two-state survival process (Fig. 1b) with

$$ {\lambda}_{02}^{\hbox{'}}(t) dt=P\left({T}^{\hbox{'}}\in \left[t,t+ dt\right)|{T}^{\hbox{'}}\ge t\right) $$

and

3 $$ {S}_0\left({t}^{\ast}\right)=\exp \left[-\left\{\underset{0}{\overset{t^{\ast }}{\int }}\lambda {\hbox{'}}_{02}(v) dv\right\}\right]. $$

Since donor availability is stochastically independent of patients’ prognosis under standard treatment, ${\lambda}_{02}(v)={\lambda}_{02}^{\hbox{'}}(v)$ until ${t}^{search}$. This independence has two important consequences. Firstly, ${S}_0\left({t}^{\ast}\right)$ is identical to the counterfactual survival probabilities in a population where there is no change in therapeutic strategies after a donor is identified, i.e. ${\lambda}_{12}(v)={\lambda}_{02}^{\hbox{'}}(v),v\ge w$; secondly, in order to estimate ${S}_0\left({t}^{\ast}\right)$ the survival information of the donor available group can be exploited up to time $w$.

### Pseudo-values

In the following the original pseudo-value approach is briefly outlined. Subsequently, generalised pseudo-values for the estimation of ${S}_0\left({t}^{\ast}\right)$ and ${S}_1\left({t}^{\ast }|w\right)$ are introduced. Furthermore, appropriate weights for ${S}_1\left({t}^{\ast }|w\right)$ are defined to compensate for partly unobserved waiting times when estimating ${S}_1\left({t}^{\ast}\right)$.

### Common pseudo-values

The pseudo-value regression technique [25–30] for censored survival data provides an attractive alternative to methods commonly applied in survival analysis. The approach specifically allows the modelling of survival probabilities at pre-specified interesting time points. Without relying on proportional hazards, pseudo-values can be flexibly regressed on common baseline covariates within the framework of a generalised linear model [15].

Let ${T}_i$ denote the time to failure and ${C}_i$ the time to censoring for the $i$-th patient, $i=1,\dots, n$. Usually ${\tilde{T}}_i=\min \left({T}_i,{C}_i\right)$ and ${D}_i=I\left({T}_i\le {C}_i\right)$ can be observed. Let $\widehat{S}(t)$ denote the ordinary Kaplan-Meier estimate and let ${\widehat{S}}^{-i}(t)$ denote the Kaplan-Meier estimate where the $i$-th observation is excluded (jacknife statistic). The pseudo-value at time ${t}^{\ast }$ [25] for the $i$-th patient is now defined as

$$ {\widehat{V}}_i\left({t}^{\ast}\right)=n\widehat{S}\left({t}^{\ast}\right)-\left(n-1\right){\widehat{S}}^{-i}\left({t}^{\ast}\right),i=1,\dots, n. $$

Individual pseudo-values can attain values below zero and above one. The approach relies on the fact that the Kaplan-Meier estimate is (approximately) unbiased for the marginal survival function [25]. Accordingly, i.i.d. observations, independent right censoring and a sufficiently large risk set at time ${t}^{\ast }$ have to be assumed. Asymptotic conditional unbiasedness of the pseudo-values given covariates can be shown [25, 32–34], which allows the use of regression models with pseudo-values as outcome (further details can be found in [25, 29]).

### Generalised 0➔2 pseudo-values for the estimation of ${S}_0\left({t}^{\ast}\right)$

For notational convenience let the first $m$ out of $n$ patients be those with an observed transition to state 1 at times ${w}_1,\dots, {w}_m$.

Let ${\widehat{S}}_0(t)$ denote the Kaplan-Meier estimate for the risk of a direct transition from state 0 to state 2 with censoring at ${w}_1,\dots, {w}_m$ of those patients with observed transitions to state 1. Hence, only direct transitions from state 0 to state 2 are considered as event. Given the assumed independence between the two stochastic processes (transitions 0→1 and 0→2), ${\widehat{S}}_0(t)$ is an asymptotically unbiased estimate of ${S}_0(t)$.

Now, for all $n$ patients 0→2 pseudo-values for ${t}^{\ast }$-year survival based on ${\widehat{S}}_0\left({t}^{\ast}\right)$ are generated according to the standard approach [25],

4 $$ {\widehat{V}}_{i,0}\left({t}^{\ast}\right)=n{\widehat{S}}_0\left({t}^{\ast}\right)-\left(n-1\right){\widehat{S}}_0^{-i}\left({t}^{\ast}\right). $$

As all patients start in state 0 and are at risk for a direct transition to state 2, it is of importance that a 0→2 pseudo-value has to be calculated for each patient. That is, patients with a transition to state 1 are censored at ${w}_1,\dots, {w}_m$ but not ignored, as the latter would lead to selection bias due to over-representing direct transitions from state 0 to state 2. Since ${\widehat{V}}_{i,0}\left({t}^{\ast}\right)$ is an asymptotically unbiasedly estimate for ${S}_0\left({t}^{\ast}\right)$, its mean ${\overline{V}}_0=\frac{1}{n}{\sum}_{i=1}^n{\widehat{V}}_{i,0}\left({t}^{\ast}\right)$ is asymptotically unbiased as well [25, 32].

### Generalised 0➔1➔2 pseudo-values for the estimation of ${S}_1\left(\left.{t}^{\ast}\right|w\right)$

To estimate ${S}_1\left(\left.{t}^{\ast}\right|{w}_i\right),i=1,\dots, m$, 0→1→2 pseudo-values have to be defined. Firstly, covering only the time after transition to state 1, 1→2 pseudo-values are calculated. For $i=1,\dots, m,$ let $\widehat{S}\left({t}^{\ast}\left|T\ge {w}_i\right.\right)$ denote the survival propability at ${t}^{\ast }$ estimated by Kaplan-Meier based on all ${n}_i$ patients still at risk at ${w}_i$. Note that ${t}^{\ast }$ is the long-term time-point of interest since time 0, and Kaplan-Meier estimates at $\left({t}^{\ast }-{w}_i\right)$ after ${w}_i$ are used. The 1→2 pseudo-value for the $i$-th patient is now defined as

5 $$ {\widehat{U}}_{i,1}\left({t}^{\ast}\left|{w}_i\right.\right)={n}_i\widehat{S}\left({t}^{\ast}\left|T\ge {w}_i\right.\right)-\left({n}_i-1\right){\widehat{S}}^{-\mathrm{i}}\left({t}^{\ast}\left|T\ge {w}_i\right.\right). $$

Here, the interval of the Kaplan-Meier estimate starts at time ${w}_i$, and accordingly the waiting time history up to ${w}_i$ can be considered as standard baseline information (see Additional file 1, Section A). Conditional on the observed waiting time ${w}_i$ for patient $i$, this pseudo-value ${\widehat{U}}_{i,1}\left({t}^{\ast}\left|{w}_i\right.\right)$ shares the properties of the original approach [25, 32–34] and

$$ E\left[{\widehat{U}}_{i,1}\left({t}^{\ast}\left|{w}_i\right.\right)\right]=S\left({t}^{\ast}\left|T\ge {w}_i,W={w}_i\right.\right)+{o}_p(1) $$

holds with $S\left({t}^{\ast}\left|T\ge {w}_i,W={w}_i\right.\right)=\underset{w_i}{\overset{t^{\ast }}{\int }}{\lambda}_{12}\left(v,v-{w}_i\right) dv$ (for details see Additional file 1, Section A).

The definition of 0→1→2 pseudo-values for ${S}_1\left({t}^{\ast }|{w}_i\right)$ requires to consider the risk of an event in state 0 until transition time ${w}_i$ as well. The Kaplan-Meier estimate ${\widehat{S}}_0\left({w}_i\right)$ can be used for this adjustment, so that the 0→1→2 generalised pseudo-value is defined by

6 $$ {\widehat{V}}_{i,1}\left({t}^{\ast}\left|{w}_i\right.\right)={\widehat{S}}_0\left({w}_i\right)\kern0.5em {\widehat{U}}_{i,1}\left({t}^{\ast}\left|{w}_i\right.\right). $$

Utilising the independence between ${\widehat{S}}_0\left({w}_i\right)$ and ${\widehat{U}}_{i,1}\left({t}^{\ast}\left|{w}_i\right.\right)$, ${\widehat{V}}_{i,1}\left({t}^{\ast}\left|{w}_i\right.\right)$ is asymptotically unbiased too,

$$ E\left[{\widehat{V}}_{i,1}\left({t}^{\ast}\left|{w}_i\right.\right)\right]={S}_0\left({w}_i\right)S\left({t}^{\ast}\left|T\ge {w}_i,W={w}_i\right.\right)+{o}_p(1)={S}_1\left({t}^{\ast}\left|{w}_i\right.\right)+{o}_p(1). $$

### Estimation of ${S}_1\left({t}^{\ast}\right)$

While the individual ${\widehat{V}}_{i,1}\left({t}^{\ast}\left|{w}_i\right.\right)$ provide an estimate of ${S}_1\left({t}^{\ast}\left|{w}_i\right.\right),i=1,\dots, m$, their mean $\frac{1}{m}\sum \limits_{i=1}^m{\widehat{V}}_{i,1}\left({t}^{\ast}\left|{w}_i\right.\right)$ estimates

7 $$ {\overline{S}}_1\left({t}^{\ast}\right)=\underset{0}{\overset{t^{\ast }}{\int }}q(w){S}_1\left({t}^{\ast}\left|w\right.\right) dw, $$

where $q(w)$ is the density of the distribution of observable waiting times before ${t}^{search}$ (Additional file 1: Section B). This density describes waiting times up to ${t}^{search}$ not prevented by the competing risks death and early censoring with the hazard functions ${\lambda}_{02}(t)$ and ${\lambda}_C(t)$, respectively. According to eq. 2, our primary aim is to estimate survival in the population with a donor, that is

$$ {S}_1\left({t}^{\ast}\right)=\underset{0}{\overset{t^{\ast }}{\int }}{f}_{01}(w)\;{S}_1\left(t|w\right) dw. $$

Note that, unless ${\lambda}_{02}(v)=0$ and ${\lambda}_C(v)=0$ for $v<{t}^{search},q(w)\ne {f}_{01}(w)$ and consequently ${\overline{S}}_1\left({t}^{\ast}\right)\ne {S}_1\left({t}^{\ast}\right)$. Since for some patients donor availability is unknown due to censoring or 0→2 transitions before time $w$, long waiting times are under-represented in $q(w)$ compared to ${f}_{01}(w)$.

In order to account for unobserved 0→1 transitions due to early 0→2 transitions and censoring, inverse probability of censoring [35] can be used to estimate the weights

8 $$ {\gamma}_{i,1}=\frac{f_{01}\left({w}_i\right)}{q\left({w}_i\right)} $$

for every observed 0→1 transition at ${w}_i,i=1,\dots, m$. As donor availability is independent of patients behaviour before ${w}_i$, all $n$ patients with and without donor available can be used. Let $\widehat{G}(w)$ denote the Kaplan-Meier estimate on the whole sample, where censoring and 0→2 transitions are considered as events and 0→1 transitions are considered as censored observations. Then

$$ E\left[\widehat{G}(w)\right]=\exp \left[-\underset{0}{\overset{w}{\int }}{\lambda}_{02}(v)+{\lambda}_C(v) dv\right]+{o}_P(1) $$

which is the probability of being uncensored and free of a 0→2 transition at time $w$. In patients with a donor available at waiting time ${w}_i$ this is equivalent to the probability $\frac{q\left({w}_i\right){p}_m}{f_{01}\left({w}_i\right)}$ of acutally observing the 0→1 transition.

The weights are then ${\widehat{\gamma}}_{i,1}=\frac{1}{\widehat{G}\left({w}_i\right)}{\widehat{p}}_m$, $i=1,\dots, m$, and ${\widehat{p}}_m=\frac{m}{\sum \limits_{j=1}^m\frac{1}{\widehat{G}\left({w}_j\right)}}$ so that $\sum \limits_{i=1}^m{\widehat{\gamma}}_{i,1}=m$.

Now, the weighted mean ${\overline{V}}_1=\frac{1}{m}\sum \limits_{i=1}^m{\widehat{\gamma}}_{i,1}{\widehat{V}}_{i,1}\left({t}^{\ast}\left|{w}_i\right.\right)$ is a consistent estimator of ${S}_1\left({t}^{\ast}\right)$ [35].

### Weighted generalised linear model for comparing ${S}_0\left({t}^{\ast}\right)$ with ${S}_1\left({t}^{\ast}\right)$

Analogous to the original pseudo-value approach, a weighted generalised linear model is utilized for inferential purposes with log-log link function, normal response probability distribution, and the parameters ${\beta}_0$ and ${\beta}_1$. Whereas ${\beta}_0$ corresponds to the intercept, ${\beta}_1$ corresponds to the binary indicator ${x}_{1i}$, with ${x}_{1i}=0$ for $i=1,\dots, n$ and ${x}_{1i}=1$ for $i=n+1,\dots, n+m$. The $\left(n+m\right)$-dimensional response vector is $\widehat{\mathbf{V}}={\left({\widehat{V}}_{1,0}\left({t}^{\ast}\right),\dots, {\widehat{V}}_{n,0}\left({t}^{\ast}\right),{\widehat{V}}_{1,1}\left({t}^{\ast}\left|{w}_1\right.\right),\dots, {\widehat{V}}_{m,1}\left({t}^{\ast}\left|{w}_m\right.\right)\right)}^{\hbox{'}}$. The weights ${\widehat{\gamma}}_{i,1}$ for ${\widehat{V}}_{i,1}\left({t}^{\ast}\left|{w}_i\right.\right)$ are defined in the previous chapter and the weights ${\widehat{\gamma}}_{i,0}$ for ${\widehat{V}}_{i,0}\left({t}^{\ast}\right)$ are set to one.

The parameter estimates are functions of the weighted means of the two types of pseudo-values; ${\widehat{\beta}}_0=g\left({\overline{V}}_0\right)$ and ${\widehat{\beta}}_1=g\left({\overline{V}}_1\right)-g\left({\overline{V}}_0\right)$. When the log-log link function is used, $g(V)=\log \left(-\log (V)\right)$ and $\exp \left({\widehat{\beta}}_1\right)$ is an asymptotically unbiased estimate of the cumulative hazard ratio at time ${t}^{\ast }$ comparing patients with and without donor available.

### Estimation of standard errors

When using the Kaplan-Meier estimate ${\widehat{S}}_0\left({w}_i\right)$ in the estimation of ${\widehat{V}}_{i,1}\left({t}^{\ast}\left|{w}_i\right.\right),i=1,\dots, m$ (eq. 6), the individual patients’ variation is not properly represented. Consequently, the weighted generalised linear model with a sandwich estimator underestimates the standard errors of $\widehat{\beta^{\hbox{'}}}=\left({\widehat{\beta}}_0,{\widehat{\beta}}_1\right)$ on average. More appropriate standard errors can be attained by replacing ${\widehat{S}}_0\left({w}_i\right)$ in eq. 6 by random draws from Bernoulli variables ${\mathrm{B}}_i$ with

$$ {\mathrm{B}}_i\sim \mathrm{Bernoulli}\left[\exp \left\{-\exp \left({p}_i\right)\right\}\right] $$ where $$ {p}_i\sim N\left(\log \left[-\log \left\{{\widehat{S}}_0\left({w}_i\right)\right\}\right],\frac{{\widehat{\sigma}}^2\left({\widehat{S}}_0\left({w}_i\right)\right)}{{\left[{\widehat{S}}_0\left({w}_i\right)\kern0.24em \log \left\{{\widehat{S}}_0\left({w}_i\right)\right\}\right]}^2}\right) $$

and ${\widehat{\sigma}}^2\left({\widehat{S}}_0\left({w}_i\right)\right)$ denotes the variance estimator of the Kaplan-Meier estimator (Greenwood’s formula). This ad-hoc approach exploits the asymptotic normality of the log-log transformation of the Kaplan-Meier estimator [31]. In practical applications, the imputations should be repeated several times and the obtained standard errors should be averaged for stability reasons.

### Software implementation

The proposed method can be straightforwardly implemented using standard routines available in the majority of statistical software packages. Details on the implementation in SAS and R are described in the Additional file 1 (Section D).

## Results

### Simulation studies

Simulation studies have been designed with ${t}^{search}={t}^{\ast }=5$ years. Survival times were generated using the inversion method [36]. Assuming survival functions from a parametric mixture cure model [21], the approach was extended to allow for a plateau of the survival curve that represents cured patients (Additional file 1: Section C). Uniform censoring times between 0 to 11 years (Table 2, Scenario A-G) and 0 to 6 years (Table 1, Scenario I and Table 2, Scenario G) were superimposed in the simulation scenarios. For each scenario, sample sizes of 400 and 1000 are investigated in 1000 simulation runs each.

**Table 1:** Results of the simulation study 1 (scenario I) using a weighted generalised linear model

			Truth			Waiting times			wGLM^k^			wGLM ad-hoc^l^		
Patients^m^	Donor	$w$	Survival S	log(−log(S))^d^	${f}_{01}(w)$	$\overline{q}(w)$ ^e^	${\overline{\gamma}}_1$ ^f^	${\widehat{f}}_{01}(w)$ ^g^	Bias^h^	SE_est_^i^	Coverage^j^	Bias^h^	SE_est_^i^	Coverage^j^
$n=1000$	No	–	0.333^a^	0.10	–	–	–	–	−0.003	0.125	95.5%	−0.003	0.125	95.5%
	Yes	–	0.620^b^	−0.74	–	–	–	–	−0.002	0.078	92.2%	−0.002	0.098	94.0%
		0.5	0.733^c^	−1.17	0.33	0.46	0.72	0.33	−0.003	0.164	94.7%	−0.002	0.172	94.0%
		1	0.681^c^	−0.96	0.33	0.39	0.86	0.33	−0.010	0.134	94.6%	−0.011	0.161	95.1%
		3	0.451^c^	−0.23	0.33	0.15	2.26	0.33	0.000	0.106	86.4%	0.004	0.191	93.7%
$n=400$ ^n^	No	–	0.333^a^	0.10	–	–	–	–	−0.004	0.210	95.6%	−0.003	0.210	95.6%
	Yes	–	0.620^b^	−0.74	–	–	–	–	−0.007	0.124	92.8%	−0.007	0.156	92.0%
		0.5	0.733^c^	−1.17	0.33	0.46	0.72	0.33	−0.024	0.263	95.4%	−0.019	0.277	95.4%
		1	0.681^c^	−0.96	0.33	0.39	0.86	0.33	−0.019	0.224	93.8%	−0.026	0.269	94.9%
		3	0.451^c^	−0.23	0.33	0.15	2.26	0.33	−0.001	0.170	85.6%	−0.017	0.309	93.4%

Weighted generalised linear model (wGLM) with log-log link; 0–6 years uniform censoring was used

^a^True survival ${S}_0(5)$ in patients without a donor available

^b^True survival ${S}_1(5)$ in patients with a donor available

^c^True survival ${S}_1\left(5\left|w\right.\right)$ in patients with a donor available at waiting time *w*

^d^log-log transformation of true survival probabilities $\mathrm{S}$

^e^Mean observed proportion of patients with a 0 → 1 transition at $w$ = 0.5, 1 and 3

^f^Mean estimated weight for $w$ = 0.5, 1 and 3

^g^Mean estimated probabilities for ${f}_{01}(w)$ at $w$ = 0.5, 1 and 3

^h^Mean difference between estimated and true $\log \left(-\log \left(\mathrm{S}\right)\right)$ values

^i^Mean of standard errors of $\log \left(-\log \left(\mathrm{S}\right)\right)$ estimates

^j^Coverage of the 95% confidence intervals for $\log \left(-\log \left(\mathrm{S}\right)\right)$

^k^The weighted generalised linear model (wGLM) uses ${\widehat{V}}_{i,1}\left({t}^{\ast}\right)$ according to eq. (6)

^l^The weighted generalised linear model (wGLM) uses the ad-hoc correction suggested to estimate ${\widehat{V}}_{i,1}\left({t}^{\ast}\right)$ (with one repetition per observation per simulation run)

^m^$n$ represents the size of the entire sample where 25% of the patients have no donor; 25% of the patients have a donor available at $w$ =0.5, 25% at $w$ =1, and 25% at $w$ =3, respectively

^n^Two of 1000 simulation runs were excluded due to non-convergence during parameter estimation

**Table 2:** Results of the simulation study 2 using a weighted generalised linear model with log-log link and ad-hoc correction of SE_est_ (with one repetition per observation per simulation run)

		Truth		Results ($n=1000$)^e^			Results ($n=400$)^e^		
Scenario	uniform censoring	${S}_0(5)$ ^a^	${\beta}_0$	Bias^b^	SE_est_^c^	Coverage^d^	Bias^b^	SE_est_^c^	Coverage^d^
A	0–11	0.404	−0.098	0.001	0.053	96.2%	0.002	0.085	95.4%
B	0–11	0.291	0.211	0.001	0.062	94.8%	0.000	0.098	95.7%
C	0–11	0.511	−0.398	0.000	0.067	94.2%	0.000	0.107	94.9%
D	0–11	0.703	−1.040	0.001	0.079	95.2%	0.000	0.126	95.2%
E	0–11	0.291	0.211	0.001	0.062	94.8%	0.000	0.098	95.7%
F	0–11	0.511	−0.398	0.000	0.067	94.2%	0.000	0.107	94.9%
G	0–11	0.333	0.095	−0.002	0.060	93.3%	−0.004	0.095	94.7%
G	0–6	0.333	0.095	−0.003	0.083	95.0%	−0.001	0.133	94.9%
		${S}_1(5)$ ^a^	${\beta}_0+{\beta}_1$	Bias^b^	SE_est_^c^	Coverage^d^	Bias^b^	SE_est_^c^	Coverage^d^
A	0–11	0.562	−0.551	−0.001	0.111	95.0%	0.000	0.177	94.9%
B	0–11	0.547	−0.505	−0.002	0.087	94.7%	0.006	0.137	93.9%
C	0–11	0.659	−0.875	−0.003	0.099	96.1%	−0.010	0.157	95.3%
D	0–11	0.703	−1.040	0.001	0.101	95.0%	0.001	0.161	95.4%
E	0–11	0.390	−0.060	0.003	0.082	95.2%	0.005	0.130	95.8%
F	0–11	0.511	−0.398	−0.006	0.087	95.0%	−0.001	0.138	94.7%
G	0–11	0.569	−0.573	−0.004	0.115	93.5%	−0.002	0.182	95.1%
G	0–6	0.569	−0.573	0.000	0.149	93.5%	−0.028	0.239	94.6%
		cHR^a^	${\beta}_1$	Bias^b^	SE_est_^c^	Coverage^d^	Bias^b^	SE_est_^c^	Coverage^d^
A	0–11	0.636	−0.453	−0.001	0.123	95.4%	−0.002	0.196	95.5%
B	0–11	0.489	−0.716	−0.004	0.107	93.8%	0.006	0.169	94.9%
C	0–11	0.621	−0.476	−0.003	0.119	95.4%	−0.010	0.190	95.3%
D	0–11	1.000	0.000	0.000	0.129	95.2%	0.001	0.204	95.4%
E	0–11	0.763	−0.271	0.001	0.103	94.8%	0.005	0.163	95.6%
F	0–11	1.000	0.000	−0.006	0.110	95.2%	−0.001	0.174	95.7%
G	0–11	0.513	−0.668	−0.003	0.131	95.0%	0.002	0.208	95.7%
G	0–6	0.513	−0.668	0.004	0.173	96.0%	−0.027	0.278	96.5%

^a^For the log-log link and ${t}^{\ast }=5:{S}_0(5)=\exp \left(-\exp \left({\beta}_0\right)\right);{S}_1(5)=\exp \left(-\exp \left({\beta}_0+{\beta}_1\right)\right);\mathrm{cHR}=\exp \left({\beta}_1\right)$

^b^Mean difference between parameter estimates and true parameters

^c^Mean standard error of the parameter estimates

^d^Coverage of the 95% confidence interval of the parameters

^e^entire sample with and without a donor

### Simulation study 1

This simulation study was performed to examine the properties of individual components of our approach, i.e. (1) the weights ${\widehat{\gamma}}_{i,1}$ to estimate ${S}_1\left({t}^{\ast}\right)$, (2) the survival estimates for ${S}_0\left({t}^{\ast}\right)$, ${S}_1\left({t}^{\ast}\left|{w}_i\right.\right)$ and ${S}_1\left({t}^{\ast}\right)$, and (3) the standard error estimates. Details of the simulation setup are provided in scenario I in Table S1 of the Additional file 1.

Seventy-five percent of all patients have a donor available. These patients equally split between discrete waiting times at $w$ = 0.5, 1 and 3 years and the according probability mass function is ${f}_{01}(w)=1/3$. The true survival probabilities and simulation results are given in Table 1.

For a sample size of 1000, the means of the observed distribution of waiting times $\widehat{q}(w)$ are 0.46, 0.39 and 0.15 for $w$ = 0.5, 1 and 3, respectively. Hence short waiting times are over- and long-waiting times are underrepresented compared to ${f}_{01}(w)$. The means of the estimated weights ${\widehat{\gamma}}_{i,1}$ are 0.72, 0.86 and 2.26 for $w$ = 0.5, 1 and 3, respectively, and the correct waiting time distribution can be restored for all $w$ with ${\widehat{f}}_{01}(w)=0.33$.

Table 1 also considers estimates from a weighted generalised linear model with a log-log link with and without the ad-hoc correction for standard error estimation. For a sample size of 1000 the bias of the $\log \left(-\log \left(\mathrm{S}\right)\right)$ estimate for both approaches is negligible with a maximum absolute value of 0.011. SE_est_ is the mean of the empirical sandwich estimates of the standard error of the $\log \left(-\log \left(\mathrm{S}\right)\right)$ estimates from the 1000 simulation runs (Table 1). Note that in the analysis of a single data set, several repeated imputations are needed to get stable standard error estimates. The mean empirical standard errors are already replicated within the 1000 simulation runs and so no repeated imputations are needed to get stable results.

Monte Carlo standard deviations are similar to mean SE_est_ and shown in Table S2 (Additional file 1). In the uncorrected case, SE_est_ tends to become smaller for increasing $w$. As expected, SE_est_ is generally larger in the ad-hoc corrected case. Consequently, with uncorrected SE_est_ estimation the coverage of the 95% confidence intervals decreases with increasing $w$ and is only 86.4% for $w=3$. When ${\widehat{V}}_{i,1}\left({t}^{\ast}\right)$ are dervied using the ad-hoc approach, the coverage substantially improves to 93.7% for ${S}_1\left(5\left|w\right.=3\right)$. The results for a sample size of 400 show a similar good performance with regard to unbiasedness of survival estimates and confidence interval coverage (Table 1).

### Stimulation study 2

This simulation study was performed to demonstrate both, approximate unbiasedness of the parameter estimates and approximate confidence interval coverage within the weighted generalised linear model with a log-log link. The standard error ad-hoc correction was used throughout in this section.

Four realistic scenarios from pediatric oncology and four theoretical scenarios are considered. Scenarios A-D are based on published results [5, 37–39] and represent realistic situations that cover all main potential types of departure from proportional hazards: differences in long-term survival only (A), crossing survival curves (B-C) and a situation where only differences in short-term survival (D) are observed. Additionally, a proportional hazards model (E), a null-model (F) and a scenario with unrealistically long-waiting times (G) were investigated. Scenario G is implemented under both, the commonly used and a much more pronounced censoring scheme, that is 0–11 and 0–6 years uniform censoring, respectively. True survival curves of patients with and without donor available are shown in Additional file 1: Figure S1 for the various scenarios together with the corresponding waiting time distributions. Details of the simulation setup are provided in scenarios A-G Table S1 and in Section C in the Additional file 1.

The results are summarised in Table 2. In scenario A, initially similar survival is observed until year 1 and afterwards stem-cell transplantation has lower hazards, leading to an inferior long-term survival probability with chemotherapy alone; ${S}_0(5)=40.4\%$ compared to ${S}_1(5)=56.2\%$. Accordingly, using the log-log link, ${\beta}_0,{\beta}_1$ and the cumulative hazard ratio (cHR) are −0.098, −0.453 and 0.636, respectively. For a sample size of 1000, the bias is 0.001 and −0.001 for ${\widehat{\beta}}_0$ and ${\widehat{\beta}}_1$, respectively. Similar small biases of 0.001 and −0.002 are seen for a sample size of 400, respectively. The mean standard errors of ${\widehat{\beta}}_0$ (SE_est_) obtained from the weighted generalised linear models are 0.053 and 0.085 for sample sizes of 1000 and 400, respectively. Furthermore, the observed coverages of the 95% confidence intervals (CI) for ${\beta}_0$ are 96.2% and 95.4%, respectively. Similar satisfying results are seen for the CI coverages of ${\beta}_1$ and ${\beta}_0+{\beta}_1$.

Likewise, convincing results are seen for Scenarios B-G as well (Table 2). In particular scenario G with uniform censoring between 0 and 6 is challenging - long waiting times coincides with heavy censoring. Even in this difficult situation, the biases of ${\widehat{\beta}}_0$ and ${\widehat{\beta}}_1$ are close to zero; the corresponding CI coverages are 95.0% and 96.0% for $n$ = 1000 and 94.9% and 96.5% for $n$ = 400, respectively.

Monte Carlo standard deviations are similar to mean SE_est_ and shown in Table S3 (Additional file 1).

In summary, in all scenarios the biases are negligibly small. Note that, the maximum bias on the scale of survival probabilities is smaller than one percentage point over all scenarios and sample sizes. The coverage probabilities of the confidence intervals are convincingly close to the nominal 95% level. As expected, the good performance of the method does not depend on the proportional hazards assumption.

### Benefit of SCT in paediatric leukaemia patients

A real dataset from a recently published international study in children with newly diagnosed Philadelphia chromosome-positive (PH+) acute lymphoblastic leukaemia [40] is used for illustrative purposes. The aim of the study is to compare SCT to conventional chemotherapy only with disease free survival (DFS) as primary endpoint. A total of 542 patients were eligible and included in the study. Of these, 217 were treated with chemotherapy only and 325 switched to SCT after a suitable donor was identified. Note that, here we only have information when an SCT was actually performed and we assume that patients received SCT immediately after their donor was identified. SCT was performed after a median waiting time of 5.1 months; the majority (95%) of SCTs were performed within 1 year.

**Fig. 2:**
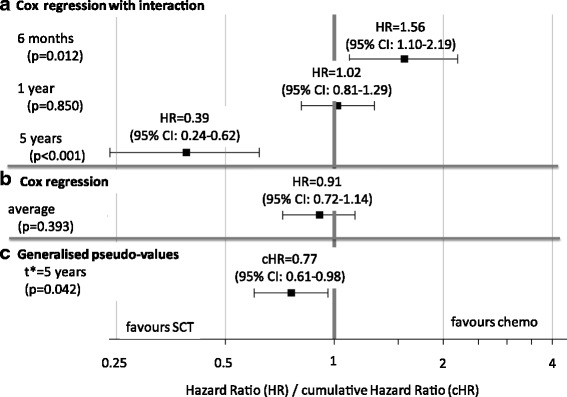
Philadelphia chromosome-positive acute lymphoblastic leukaemia: results of Cox regression with a binary time-dependent covariate with/without log(time) interaction versus the generalised pseudo-value approach (no adjustment for baseline covariates)

The application of the generalised pseudo-values approach is contrasted to the popular Cox model. The original analysis allowed for non-proportional hazards [40] by including a time-dependent treatment indicator and its interaction with log(time) in a Cox regression model. The hazard ratios at 6 months and 5 years were estimated to be 1.56 and 0.39, respectively (Fig. 2). While there is a clear evidence of lower hazards with SCT at later time points, the average hazard ratio of 0.91 (*p* = 0.39) from a Cox regression model with a time-dependent treatment indicator shows little evidence of a beneficial SCT effect (Fig. 2). However due to the ignorance of the non-proportional hazards these results may be overly sensitive to the initial disadvantage of SCT and may understate the positive impact of SCT in the long term.

For 5-years DFS, the generalisation of pseudo-value regression for a time-dependent covariate is applied with a log-log link and ${t}^{search}={t}^{\ast }=5$ years. The log-cumulative hazard ratio is −0.26 and the estimated 5-year cHR is 0.77. Using the ad-hoc approach as described in subsection ‘Estimation of standard errors’ with 1000 repeated imputations, the mean standard error of the log-cumulative hazard ratio is 0.123. The corresponding 95% confidence interval for cHR is 0.61 to 0.98 (Fig. 2) showing a positive cumulative effect of SCT at 5 years with a Wald-type *p*-value of 0.042. This cHR is directly related to the 5-year DFS probability estimates of 42% and 32% with and without donor, respectively.

## Discussion

Unresolved methodological challenges when comparing long-term survival probabilities with and without stem-cell transplantation are the motivation of this work. Although the medical interest is in assessing the effect of this time-dependent intervention, the comparison has to be based on donor availability. Firstly, donor availability is an external process defining two populations and allowing proper estimation and interpretation of survival probabilities. Secondly, as donor availability is independent of patient’s prognosis, patients with and without donor available are comparable before the intervention so that selection bias can be ruled out. On the contrary, if donor availability status is not properly documented and donor identification does not immediately or not at all lead to an SCT then a selection bias might occur and the results of any statistical analysis may lack sensible interpretation.

If for some patients donor availability is unknown due to early death or censoring before ${t}^{search}$, a simple comparison according to donor availability is not possible. In such situations, Cox-regression with a binary time-dependent covariate is often considered as a seemingly obvious choice. However, severe violations of the proportional hazards assumption renders the results of such an analysis useless. Even if the time-varying hazard ratio is appropriately modelled, the results of a Cox model do not clearly show, whether the long-term intervention benefit is able to outweigh the short-term intervention risks.

In contrast, the generalised pseudo-values approach investigates the primarily interesting cumulative hazards in both populations up to a long-term time point of interest ${t}^{\ast }$. Now, allowing for incomplete information on donor availability and without relying on proportional hazards, the generalised pseudo-value approach provides a direct comparison and helps to decide whether the benefits of SCT justifies its therapeutic use in future patients.

Due to the presence of long-term survivors and cured individuals in paediatric oncology, the choice of an appropriate long-term time point ${t}^{\ast }$ is usually straightforward. In other situations, in particular when cure of patients is less common, the choice of a single time point ${t}^{\ast }$ might be less obvious; a simultaneous investigation of several time points may then provide a more complete picture analogous to the original pseudo-value approach.

Finally, note that a further investigation of the association between waiting times and 0→1→2 pseudo-values could provide an answer to the clinical research question, whether a late identified donor should still lead to an SCT. Additionally, the generalised pseudo-values method can be adapted to include (baseline) covariates. These aspects will be investigated in future work.

## Conclusion

Mimicking a randomised comparison, the proposed generalisation of the pseudo-value regression technique enables to compare survival probabilities in patients with and without a donor, although donor availability is incompletely observed. A clinically relevant but methodologically difficult situation can now be reasonably addressed with results that are reliable and easy to communicate.

## Additional files

Additional file 1: **Section A.** Properties of the 1 → 2 pseudo-value. **Section B.** Waiting time distribution in patients with a donor. **Section C.** Generation of simulated data. **Section D.** Software implementation (PDF 396 kb)
