# Supplementary material for: Assessing the effect of a partly unobserved, exogenous, binary time-dependent covariate on survival probabilities using generalised pseudo-values
Source: BMC Med Res Methodol. 2018 Jan 19;18:14. doi: 10.1186/s12874-017-0430-5 (PMC5775686; doi:10.1186/s12874-017-0430-5)
Supplement: Supplementary file 1 — Section A. Properties of the 1 → 2 pseudo-value. Section B. Waiting time distribution in patients with a donor. Section C. Generation of simulated data. Section D. Software implementation (PDF 396 kb) [file 12874_2017_430_MOESM1_ESM.pdf]

Assessing the effect of a partly unobserved, exogenous, binary time-dependent covariate on survival probabilities using generalised pseudo-values

Ulrike Pötschger[1,2], Harald Heinzl[2], Maria Grazia Valsecchi[3], Martina Mittlböck[2*]

-APPENDIX-

Section A: Properties of the 1→2 pseudo-value

Consider a value z , $0 \le z \le t^{search}$, and let $\hat{S}\left(t^* \middle| T \ge z\right)$ denote the survival propability at t^* estimated by Kaplan-Meier based on all r patients still at risk at z . The common pseudo-value [1] for the *k-th* patient, $k = 1 \ldots r$, is now defined as

$$\hat{U}_k = r\hat{S}\left(t^* \middle| T \ge z\right) - (r-1)\hat{S}^{-k}\left(t^* \middle| T \ge z\right). \tag{1}$$

Let z_k denote the waiting time of patient k which is set to, say, $z+1$, for patients in state 0 at z . Now $Z_k = \left(\min\left(z_k, z\right), \mathrm{I}\left(z_k \le z\right)\right)$ is the waiting time history of patient k up to time z . Since Z_k is a vector of baseline variables at z the arguments with respect to the asymptotic properties of the pseudo-values of Jacobsen and Martinussen [2] apply in analogy.

Now $\left(\left(\tilde{T}_k - z, D_k\right), Z_k\right)$, $k = 1 \ldots r$, can be considered as i.i.d. replicates of the population at risk at z . Using the von Mises expansion the pseudo-value can be expressed as

$$\hat{U}_k = \theta + \dot{\psi}\left(\tilde{T}_k - z, D_k\right) + o_p(1)$$

where $\theta = P\left(T > t^* \middle| T \ge z\right) = S\left(t^* \middle| T \ge z\right)$ denotes the survival probabiltiy at t^* of the population at risk at z ; and $\hat{S}\left(t^* \middle| T \ge z\right)$ is an asymptotically unbiased estimate of θ.

Jacobson and Martinussen [2] showed that

$$E\left[\dot{\psi}\left(\tilde{T}_k - z, D_k\right)\middle|Z_k\right] = \theta_k - \theta .$$

Here, $\theta_k = P\left(T_k > t^* \middle| T_k \geq z, Z_k\right) = S\left(t^* \middle| T \geq z, Z_k\right)$ is the survival probability at t^* conditional on both, being alive at z and the waiting time history of patient k up to z .

Consequently it follows that

$$E\left[\hat{U}_k\right] = \theta_k + o_p(1).$$

By varying z between 0 and t^{search}, a plethora of pseudo-values could be computed. However, the primary interest is in values of z that correspond to actually observed waiting times w_i, $i = 1\ldots m$. Given $z = w_i$ and a patient with $z_k = w_i$, then

$$\theta_k = S\left(t^* \middle| T \geq w_i, W = w_i\right) = \int_{w_i}^{t^*} \lambda_{12}\left(v, v - w_i\right) dv$$

which is the quantity to be estimated in the main paper.

Section B: Waiting time distribution in patients with a donor

Here, the density $f_{01}(w)$ of partly unobservable times to donor identification (waiting times) in patients with a donor available is related to the density $q(w)$ of observable waiting times up to t^{search} not prevented by competing risks, like death and early censoring represented by $\lambda_{02}(t)$ and $\lambda_C(t)$, respectively. Now,

$$q(w) = \frac{1}{p_m} \lambda_{01}(w) \exp\left[-\int_0^w \lambda_{01}(v) + \lambda_{02}(v) + \lambda_C(v) dv\right]$$

with $p_m = \int_0^{t^{search}} \lambda_{01}(x)\exp\left[-\int_0^x \lambda_{01}(v)+\lambda_{02}(v)+\lambda_C(v)dv\right]dx$, so that $\int_0^{t^{search}} q(w)dw = 1$. Here, p_m is the expected proportion of patients with observed 0→1 transition in the population of patients with a donor available. Due to the competing risks $\lambda_{02}(t)$ and $\lambda_C(t)$, longer waiting times are underrepresented among the *m* patients with observed 0→1 transitions.

For the estimation of $S_1(t^*)$, the density $f_{01}(w)$ of times to donor identification of all patients with a donor available (includes patients with ceased donor search) is needed. This quantity is

$$f_{01}(w) = \lambda_{01}(w)\exp\left[-\int_0^w \lambda_{01}(v)dv\right]$$

and it is linked to $q(w)$ by

$$f_{01}(w) = \frac{q(w)\ p_m}{\exp\left[-\int_0^w \lambda_{02}(v)+\lambda_C(v)dv\right]}.$$

Given *W*=*w*, the denominator represents the probability that a 0→1 transition can actually be observed at time *w*.

Section C: Generation of simulated data

Section C is concerned with the generation of simulated survival and waiting times. Parameter values used in the simulations can be found in Table S1.

Let $S_{Wb}(t;\omega,\vartheta) = \exp(-\omega t^{\vartheta})$ denote the survival function of a Weibull distribution for t≥0, where $\vartheta > 0$ is the shape parameter and $\omega > 0$ represents the scale parameter. Let $f_{Wb}(t;\omega,\vartheta)$ denote the corresponding density distribution.

For direct 0→2 transitions, the simulations are based on the parametric mixture survival function

$S_0(t)=\pi_{02}S_{Wb}(t;\omega_{02},\vartheta_{02})+(1-\pi_{02})$.

Here, $(1-\pi_{02})$ is the proportion of cured patients and $S_0(t)$ converges to $(1-\pi_{02})$ with increasing *t*. This mimics the typical situation in paediatric oncology where the plateau in the survival function indicates the presence of long-term survivors (cured patients). π_{02} is the proportion of patients that are susceptible for a direct 0→2 transition with corresponding survival function $S_{Wb}(t;\omega_{02},\vartheta_{02})$. Until $t^{search}=t^*=5$ years, the hazard functions for a 0→2 transition are

$\lambda_{02}(t)=\lambda'_{02}(t)=\dfrac{\pi_{02}f_{Wb}(t;\omega_{02},\vartheta_{02})}{S_0(t)}$ in the populations with and without donor available,

respectively. More details on parametric mixture survival function can be found in Sposto [4].

Additionally, times to 0→1 transitions (waiting times *w*) need to be simulated. It is assumed that a proportion of π_{01} patients have a donor available. For scenarios A-G, a log-normal waiting time distribution $f_{01}(w)$ with parameters μ_{01} and σ_{01}, truncated at t^{search}, is used. The cumulative density function of the log-normal distribution is for x>0

$$CDF(x,\mu,\sigma)=\frac{1}{\sigma\sqrt{2\pi}}\int_{-\infty}^{\log(x)} e^{\left[-\frac{(v-\mu)^2}{2\sigma^2}\right]}dv$$

In scenario I, discrete waiting times at w=0.5, 1 and 3 years and the probability mass function $f_{01}(w)=1/3$ are assumed.

For the population with a donor available and for a specific waiting time $w \leq t$, the following form of the hazard function for a 1→2 transition was used in the simulations:

$$\lambda_{12}(t,t-w)=r\lambda_{02}(t)+\lambda_T(t-w)$$

Here $\lambda_{12}(t,t-w)$ depends on both, the time elapsed since time zero and the time elapsed since the 0→1 transition at time w. The first term, $r\lambda_{02}(t)$, represents the long-term effect of the time-dependent intervention, which is favourable when r<1. The second term, $\lambda_T(t-w)$, allows for additional short-term risks due to the intervention and follows a Weibull mixture distribution $S_T(t-w)=\pi_T S_{Wb}(t-w;\omega_T,\vartheta_T)+(1-\pi_T)$. The proportion π_T represents the specific intervention related events in state 1 that would be observed in absence of any other competing events.

Section D: Software implementation

The proposed method can be straightforwardly implemented using standard routines available in the majority of statistical software packages. Firstly, Kaplan-Meier estimates are repeatedly computed to derive generalised pseudo-values; subsequently, a generalised linear model is fitted.

In SAS, the procedure LIFETEST provides Kaplan-Meier estimates for survival probabilities. The procedure GENMOD can be used for fitting a generalised linear model. Note, that the model specification is done identically to the original pseudo-value approach, e.g. see Klein et al. [5] for details.

In R, the function ‘survfit’ in the package SURVIVAL can be used for Kaplan-Meier estimates. The generalised linear model can be estimated using the object ‘geese’ in the package GEEPACK. For a more detailed description of the technical implementation see Klein et al. [5].

Additional file 1: Table S1: Specification of the simulated scenarios: parameter values and 'true' 5-year survival probabilities

Scenario		Survival times							Waiting times[1]			True survival probabilities[2]	
		Transition 0→2			Transition 1→2				Transition 0→1				
		$1-\pi_{02}$	ω_{02}	ϑ_{02}	r	π_T	ω_T	ϑ_T	π_{01}	μ_{01}	σ_{01}	$S_0(5)$	$S_1(5)$
I	Discrete	0.18	0.150	1.5	0.1	0.15	3	1.3	0.75	-	-	0.333	0.620
A	Balduzzi 2005	0.4	0.629	1.3	0.33	0.18	8.5	2.5	0.25	log(0.4)	0.3	0.404	0.562
B	Gale 1998	0.18	0.179	1.5	0.1	0.35	3	1.3	0.4	log(0.5)	0.3	0.291	0.547
C	Goldstone 2008	0.5	0.210	1.8	0.3	0.16	10	1.5	0.4	log(0.7)	0.3	0.511	0.659
D	Locaciulli 2007	0.7	0.653	1.2	0.4	0.16	4	2.5	0.4	log(0.4)	0.3	0.703	0.703
E	PH	0.18	0.179	1.5	0.75	0	-	-	0.4	log(0.5)	0.3	0.291	0.390
F	No diff.	0.5	0.210	1.8	1	0	-	-	0.4	log(0.7)	0.3	0.511	0.511
G	Late SCTs	0.18	0.150	1.5	0.1	0.15	3	1.3	0.45	log(2)	0.8	0.333	0.569

1) $t^{search}=t^*=5$ years

2) True survival probabilities $S_0(5)$ and $S_1(5)$ were calculated using computations and simulations in SAS and numerical integration in Mathematica according to equation (3) and (2) of the main paper, respectively.

Additional file 1: Table S2: Model-based and Monte-Carlo standard-errors for simulation study 1

			**wGLM[1]**		**WGLM ad-hoc[2]**	
	**Donor**	**w**	**SE_{est}[3]**	**SD_{sim}[4]**	**SE_{est}[3]**	**SD_{sim}[4]**
n=1000[5]	No Donor	--	0.125	0.128	0.125	0.128
	Yes	--	0.078	0.070	0.098	0.104
		0.5	0.164	0.158	0.172	0.168
		1	0.134	0.132	0.161	0.158
		3	0.106	0.083	0.191	0.177
n=400[5]	No Donor	--	0.210	0.216	0.210	0.216
	Yes	--	0.124	0.112	0.156	0.171
		0.5	0.263	0.253	0.277	0.269
		1	0.224	0.211	0.269	0.254
		3	0.170	0.129	0.309	0.286

1) The weighted generalised linear model (wGLM) uses $\hat{V}_{i,1}(t*)$ according to equation (6)
2) The weighted generalised linear model (wGLM) uses the ad-hoc correction suggested to estimate $\hat{V}_{i,1}(t*)$ (with one repetition per observation per simulation run)
3) Mean of standard errors of the generalised linear model (empirical 'sandwich' estimator)
4) Standard deviations of the parameter estimates of 1000 simulation runs (Monte-Carlo standard deviations)
5) Entire sample with and without a donor with 25 % in every subgroup: without donor and donor found at *w*=0.5, 1 and 3, respectively

Additional file 1: Table S3: Model-based and Monte-Carlo standard-errors for simulation study 2

β_0	uniform censoring	n=1000[3] SE_{est}[1]	n=1000[3] SD_{sim}[2]	n=400[3] SE_{est}[1]	n=400[3] SD_{sim}[2]
**A**	0-11	0.053	0.051	0.085	0.082
**B**	0-11	0.062	0.063	0.098	0.095
**C**	0-11	0.067	0.068	0.107	0.107
**D**	0-11	0.079	0.080	0.126	0.126
**E**	0-11	0.062	0.062	0.098	0.095
**F**	0-11	0.067	0.068	0.107	0.107
**G**	0-11	0.060	0.063	0.095	0.097
**G**	0-6	0.083	0.083	0.133	0.132
$\beta_0+\beta_1$					
**A**	0-11	0.111	0.111	0.177	0.181
**B**	0-11	0.087	0.092	0.137	0.142
**C**	0-11	0.099	0.098	0.157	0.154
**D**	0-11	0.101	0.105	0.161	0.162
**E**	0-11	0.082	0.081	0.130	0.129
**F**	0-11	0.087	0.087	0.138	0.142
**G**	0-11	0.115	0.125	0.182	0.187
**G**	0-6	0.149	0.154	0.239	0.246
β_1					
**A**	0-11	0.123	0.122	0.196	0.196
**B**	0-11	0.107	0.112	0.169	0.173
**C**	0-11	0.119	0.118	0.190	0.184
**D**	0-11	0.129	0.127	0.204	0.201
**E**	0-11	0.103	0.101	0.163	0.161
**F**	0-11	0.110	0.109	0.174	0.174
**G**	0-11	0.131	0.132	0.208	0.204
**G**	0-6	0.173	0.169	0.278	0.268

1) Mean of standard errors of the generalised linear model (empirical 'sandwich' estimator)
2) Standard deviations of the parameter estimates of 1000 simulation runs (Monte-Carlo standard deviations)
3) entire sample with and without a donor

Additional file 1: Figure S1: Survival scenarios used in simulation study 2 with t^{search}=5 years

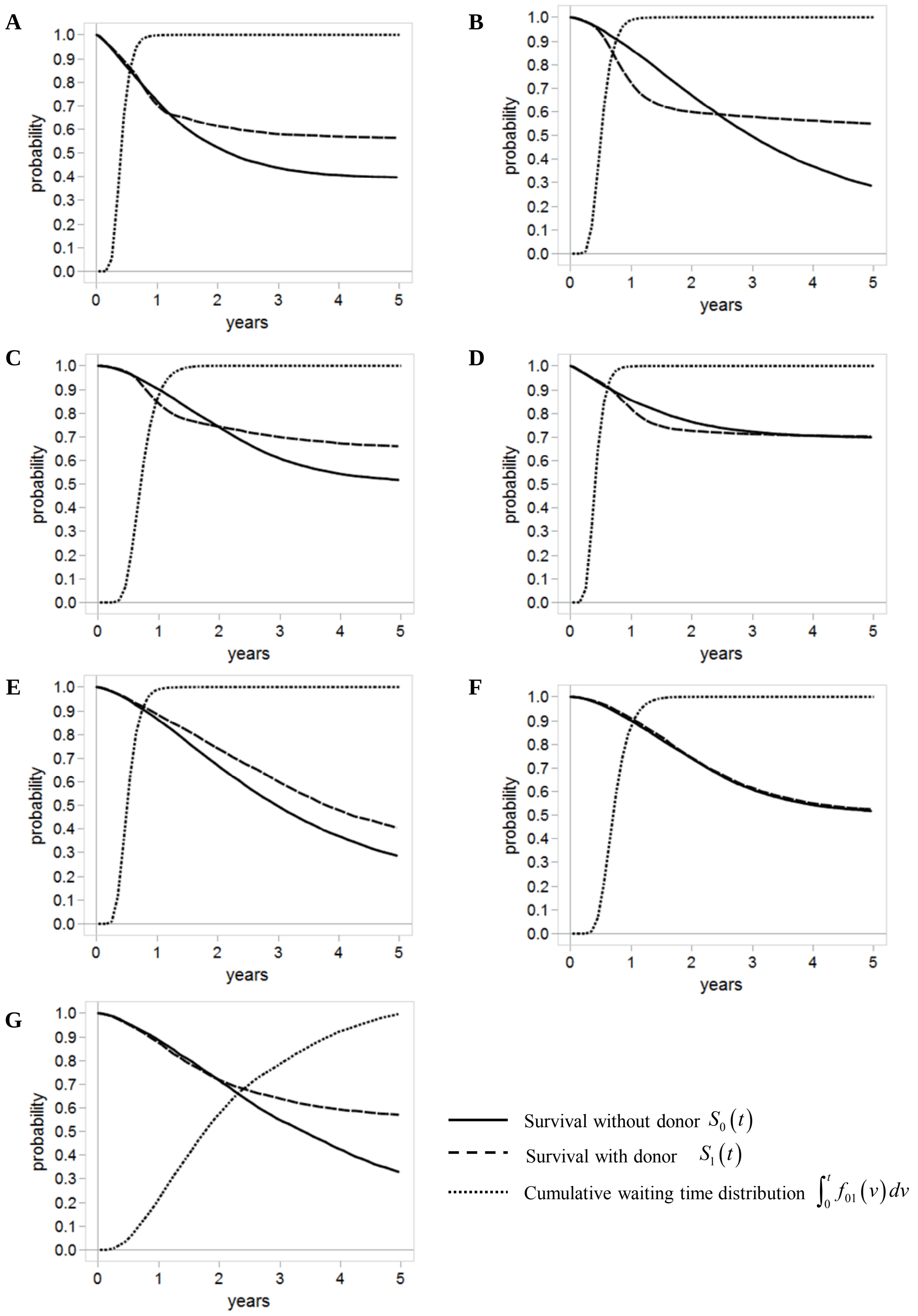

References

1. Andersen PK, Pohar Perme M: **Pseudo-observations in survival analysis**. *Statistical methods in medical research* 2010, **19**(1):71-99.
2. Jacobsen M, Martinussen T: **A Note on the Large Sample Properties of Estimators Based on Generalized Linear Models for Correlated Pseudo-observations**. *Scandinavian Journal of Statistics* 2016, **43**(3):845-862.
3. Rotnitzky A, Robins JM: **Inverse Probability Weighting in Survival Analysis**. In: *Encyclopedia of Biostatistics.* Edited by Armitage P, Colton T: John Wiley; 2005.
4. Sposto R: **Cure model analysis in cancer: an application to data from the Children's Cancer Group**. *Statistics in Medicine* 2002, **21**(2):293-312.
5. Klein JP, Gerster M, Andersen PK, Tarima S, Pohar Perme M: **SAS and R functions to compute pseudo-values for censored data regression**. *Computer Methods and Programs in Biomedicine* 2008, **89**(3):289-300.
